# Supplementary material for: Direct and indirect nutritional factors that determine reproductive performance of heifer and primiparous cows
Source: PLoS One. 2022 Oct 4;17(10):e0275426. doi: 10.1371/journal.pone.0275426 (PMC9531839; doi:10.1371/journal.pone.0275426)
Supplement: Attachment — Submitted filename: PONE-D-21-30153_revAE.pdf [file pone.0275426.s002.pdf]

PLOS ONE

Direct and indirect nutritional factors that determine reproductive performance of heifer and primiparous cows

--Manuscript Draft--

**Manuscript Number:**	PONE-D-21-30153
**Article Type:**	Research Article
**Full Title:**	Direct and indirect nutritional factors that determine reproductive performance of heifer and primiparous cows
**Short Title:**	Factors that determine reproductive performance of beef cows
**Corresponding Author:**	Lidiane Raquel Eloy<br>Federal University of Rio Grande do Sul: Universidade Federal do Rio Grande do Sul<br>Porto Alegre, RS BRAZIL
**Keywords:**	body weight at the beginning of the breeding season; breeding season; pasture type; pregnancy rate; stocking rate
**Abstract:**	Pregnancy rate is a major determinant of population dynamics of wild ungulates and of productivity of livestock systems. Allocation of feeding resources, including stocking rates, prior to and during the breeding season is a crucial determinant of this vital rate. Thus, quantification of effects and interaction among multiple factors that affect pregnancy rate is essential for management and conservation of pasture-based systems. Pregnancy rate of 2982 heifers and primiparous cows was studied as a function of animal category, average daily gain during the breeding season, stocking rate, pasture type and body weight at the beginning of the breeding season. Data were obtained from 43 experiments conducted in commercial ranches and research stations in the Pampas region between 1976 and 2015. Stocking rate ranged from 200 to 464 kg live weight ha $^{-1}$, which brackets values for most of the grazinglands in similar regions. Age at breeding was 14-36 months (24.6±7.5 months); initial breeding weights were 129-506 kg and 194-570 kg for heifers and primiparous cows. Pregnancy rate was modeled with an apriori set of explanatory variables where proximate variables (breed, body weight at start of breeding, weight gain during breeding and category) were included first and subsequently modeled as functions of other variables (pasture type, supplementation and stocking rate). This modeling approach allowed detection of direct and indirect effects (through nutrition and body weight) of factors that affect pregnancy rate. Taurine (*Bos taurus* breeds, N = 1058) had higher pregnancy rate than *B. Taurus* x *B. indicus* crossbreed (N = 1924) females.<br>Pregnancy rate of heifers and primiparous cows grazing in natural grasslands decreased with increasing stocking rate, but no effect of stocking rate was detected in cultivated and improved pastures. Pregnancy rate increased with increasing average daily gain during the breeding season. Use of cultivated or improved natural pastures promotes higher pregnancy rate, as well as allows higher in stocking rate at the regional level. Body weight at the start of the breeding season is the primary determinant of pregnancy rates in heifer and primiparous cows.
**Order of Authors:**	Lidiane Raquel Eloy<br>Carolina Bremm<br>José Fernando Piva Lobato<br>Luciana Pötter<br>Emilio A Laca
**Opposed Reviewers:**	
**Additional Information:**	
**Question**	**Response**
**Financial Disclosure**	Funding for this research was provided by grants from the Brazilian National Council for Scientific and Technological Development (CNPq) and CAPES.

Powered by Editorial Manager® and ProduXion Manager® from Aries Systems Corporation

Enter a financial disclosure statement that describes the sources of funding for the work included in this submission. Review the submission guidelines for detailed requirements. View published research articles from *PLOS ONE* for specific examples.<br><br>This statement is required for submission and **will appear in the published article** if the submission is accepted. Please make sure it is accurate.<br><br>**Unfunded studies**<br>Enter: *The author(s) received no specific funding for this work.*<br><br>**Funded studies**<br>Enter a statement with the following details:<br>• Initials of the authors who received each award<br>• Grant numbers awarded to each author<br>• The full name of each funder<br>• URL of each funder website<br>• Did the sponsors or funders play any role in the study design, data collection and analysis, decision to publish, or preparation of the manuscript?<br>• **NO** - Include this sentence at the end of your statement: *The funders had no role in study design, data collection and analysis, decision to publish, or preparation of the manuscript.*<br>• **YES** - Specify the role(s) played.<br><br>* typeset	
**Competing Interests**<br><br>Use the instructions below to enter a competing interest statement for this submission. On behalf of all authors, disclose any competing interests that could be perceived to bias this work—acknowledging all financial support and any other relevant financial or non-financial competing interests.<br><br>This statement is required for submission and **will appear in the published article** if the submission is accepted. Please make	No authors have competing interests.

sure it is accurate and that any funding sources listed in your Funding Information later in the submission form are also declared in your Financial Disclosure statement.<br><br>View published research articles from *PLOS ONE* for specific examples.<br><br>**NO authors have competing interests**<br><br>Enter: *The authors have declared that no competing interests exist.*<br><br>**Authors with competing interests**<br><br>Enter competing interest details beginning with this statement:<br><br>*I have read the journal's policy and the authors of this manuscript have the following competing interests: [insert competing interests here]*<br><br>* typeset	
**Ethics Statement**<br><br>Enter an ethics statement for this submission. This statement is required if the study involved:<br><br>• Human participants<br>• Human specimens or tissue<br>• Vertebrate animals or cephalopods<br>• Vertebrate embryos or tissues<br>• Field research<br><br>Write "N/A" if the submission does not require an ethics statement.<br><br>General guidance is provided below. Consult the submission guidelines for detailed instructions. **Make sure that all information entered here is included in the Methods section of the manuscript.**	"N/A"

**Format for specific study types**<br><br>**Human Subject Research (involving human participants and/or tissue)**<br>• Give the name of the institutional review board or ethics committee that approved the study<br>• Include the approval number and/or a statement indicating approval of this research<br>• Indicate the form of consent obtained (written/oral) or the reason that consent was not obtained (e.g. the data were analyzed anonymously)<br><br>**Animal Research (involving vertebrate animals, embryos or tissues)**<br>• Provide the name of the Institutional Animal Care and Use Committee (IACUC) or other relevant ethics board that reviewed the study protocol, and indicate whether they approved this research or granted a formal waiver of ethical approval<br>• Include an approval number if one was obtained<br>• If the study involved *non-human primates*, add *additional details* about animal welfare and steps taken to ameliorate suffering<br>• If anesthesia, euthanasia, or any kind of animal sacrifice is part of the study, include briefly which substances and/or methods were applied<br><br>**Field Research**<br><br>Include the following details if this study involves the collection of plant, animal, or other materials from a natural setting:<br>• Field permit number<br>• Name of the institution or relevant body that granted permission	
**Data Availability**<br><br>Authors are required to make all data underlying the findings described fully available, without restriction, and from the time of publication. PLOS allows rare exceptions to address legal and ethical concerns. See the PLOS Data Policy and FAQ for detailed information.	Yes - all data are fully available without restriction

A Data Availability Statement describing where the data can be found is required at submission. Your answers to this question constitute the Data Availability Statement and **will be published in the article**, if accepted.<br><br>**Important:** Stating 'data available on request from the author' is not sufficient. If your data are only available upon request, select 'No' for the first question and explain your exceptional situation in the text box.<br><br>Do the authors confirm that all data underlying the findings described in their manuscript are fully available without restriction?	
**Describe where the data may be found in full sentences. If you are copying our sample text, replace any instances of XXX with the appropriate details.**<br><br>• If the data are **held or will be held in a public repository**, include URLs, accession numbers or DOIs. If this information will only be available after acceptance, indicate this by ticking the box below. For example: *All XXX files are available from the XXX database (accession number(s) XXX, XXX.*).<br>• If the data are all contained **within the manuscript and/or Supporting Information files**, enter the following: *All relevant data are within the manuscript and its Supporting Information files.*<br>• If neither of these applies but you are able to provide **details of access elsewhere**, with or without limitations, please do so. For example:<br><br>*Data cannot be shared publicly because of [XXX]. Data are available from the XXX Institutional Data Access / Ethics Committee (contact via XXX) for researchers who meet the criteria for access to confidential data.*<br><br>*The data underlying the results presented in the study are available from (include the name of the third party*	Data would be available in supplementary file.

and contact information or URL).<br>• This text is appropriate if the data are owned by a third party and authors do not have permission to share the data.<br><br>* typeset	
Additional data availability information:	Tick here if the URLs/accession numbers/DOIs will be available only after acceptance of the manuscript for publication so that we can ensure their inclusion before publication.

Powered by Editorial Manager® and ProduXion Manager® from Aries Systems Corporation

UNIVERSITY OF CALIFORNIA, DAVIS

BERKELEY • DAVIS • IRVINE • LOS ANGELES • MERCED • RIVERSIDE • SAN DIEGO • SAN FRANCISCO   SANTA BARBARA • SANTA CRUZ

DEPARTMENT OF PLANT SCIENCES
MAIL STOP 1
UNIVERSITY OF CALIFORNIA
ONE SHIELDS AVE
DAVIS, CALIFORNIA 95616-8780
TELEPHONE: 530-752-1703
FAX: 530-752-4361

COLLEGE OF AGRICULTURAL AND
ENVIRONMENTAL SCIENCES
AGRICULTURAL EXPERIMENT STATION
COOPERATIVE EXTENSION

17 September 2021

Dear Editor,

Please, consider the attached manuscript entitled "Direct and indirect nutritional factors that determine reproductive performance of heifer and primiparous cows", for publication in the Plos One Journal. The corresponding author is Dr. Lidiane R. Eloy (lidianeloy@hotmail.com).

Our manuscript focuses on the multiple studies of factors that affect pregnancy rates under production conditions in the Pampas region of Southern Brazil, including stocking rate. Stocking rate is a primary factor in grazing management, but its study is rare. Our article is based on merged raw data from 43 experiments conducted over multiple sites. Most of these experiments were never published before.

We quantified the effects of stocking rate, body weight at the beginning of the breeding season, changes in body weight during the breeding season, animal category, breed, type of pasture and supplementation before and during breeding. Results show that the most important factor that influenced the pregnancy rate is body weight at the beginning of the breeding season. The second factor that influenced the pregnancy rate is change in body weight per day during the breeding season. Interestingly, stocking rate had direct effects on pregnancy rate that were not accounted for by body weight or change in body weight. Animal category and pasture at the beginning of the breeding season influenced the pregnancy rate.

This manuscript is original. The work is part of the PhD thesis of the first author and it is not being considered for publication anywhere. The manuscript has been approved by all co-authors. The authors declare they have no conflict of interest.

Yours sincerely,

Lidiane Eloy

Lidiane R. Eloy

Emilio Laca

Emilio A. Laca
Professor

Manuscript Click here to access/download;Manuscript;Manuscript.docx

Direct and indirect nutritional factors that determine reproductive performance of heifer and primiparous cows[1]

Nutritional factors determining performance of heifers and cows

Lidiane R. Eloy*[1], Carolina Bremm[1], José F. P. Lobato[1], Luciana Pötter[2], Emilio A. Laca[3]

[1]Faculty of Agronomy, Animal Science Department, Federal University of Rio Grande do Sul, Porto Alegre, Rio Grande do Sul, Brazil

[2]Rural Science Center, Animal Science Department, Federal University of Santa Maria, Santa Maria, Rio Grande do Sul, Brazil

[3]Department of Plant Sciences, University of California, Davis, California, United States of America

*Corresponding author

E-mail: lidianeloy@hotmail.com

Abstract

Pregnancy rate is a major determinant of population dynamics of wild ungulates and of productivity of livestock systems. Allocation of feeding resources, including stocking rates, prior to and during the breeding season is a crucial determinant of this vital rate. Thus, quantification of effects and interaction among multiple factors that affect pregnancy rate is essential for management and conservation of pasture-based systems. Pregnancy rate of 2982 heifers and primiparous cows was studied as a function of animal category, average daily gain during the breeding season, stocking rate, pasture type and body weight at the beginning of the breeding season. Data were obtained from 43 experiments conducted in commercial ranches and research stations in the Pampas region between 1976 and 2015. Stocking rate ranged from 200 to 464 kg live weight ha^{-1}, which brackets values for most of the grazinglands in similar regions. Age at breeding was 14-36 months (24.6±7.5 months); initial breeding weights were 129-506 kg and 194-570 kg for heifers and primiparous cows. Pregnancy rate was modeled with an apriori set of explanatory variables where proximate variables (breed, body weight at start of breeding, weight gain during breeding and category) were included first and subsequently modeled as functions of other variables (pasture type, supplementation and stocking rate). This modeling approach allowed detection of direct and indirect effects (through nutrition and body weight) of factors that affect pregnancy rate. Taurine (*Bos taurus* breeds, N = 1058) had higher pregnancy rate than *B. Taurus* x *B. indicus* crossbreed (N = 1924) females.

Pregnancy rate of heifers and primiparous cows grazing in natural grasslands decreased with increasing stocking rate, but no effect of stocking rate was detected in cultivated and improved pastures. Pregnancy rate increased with increasing average daily gain during

the breeding season. Use of cultivated or improved natural pastures promotes higher pregnancy rate, as well as allows higher ~~in~~ stocking rate at the regional level. Body weight at the start of the breeding season is the primary determinant of pregnancy rates in heifer and primiparous cows.

Introduction

Although fundamental cattle physiology and its relation to nutrition are well known, there is not sufficient quantitative information about how management affects nutrition and reproduction, especially under natural grasslands conditions. In particular, few studies have been done to evaluate the relative effects of stocking rate and other nutritional management factors on pregnancy rates at a regional level (Gottschall & Lobato 1996; Simeone & Lobato 1996; Quadros & Lobato 1996; Fagundes, Lobato & Schenkel 2003; Pötter *et al*. 2010; Sartori & Guardieiro 2010). The lack of single studies with regional scope is understandable because such studies with livestock are logistically complex and extremely expensive.

An alternative to specific comprehensive studies is to analyze data pooled form multiple studies (Duffield, Merril & Bagg 2012; Lean, Thompson & Dunshea 2014) that address the same research question using equivalent response and explanatory variables, that is, a meta-analysis (Schwarzer, Carpenter & Rücker 2015). Meta-analysis requires care in the process of systematizing results from multiple studies but it has the advantage of increasing precision, decreasing costs and research time, and increasing the degrees of freedom in the analysis (Fisher 1999; Lovatto, Lehnen, Andretta, Carvalho & Hauschild 2007).

Livestock production in the Pampas region of Southern Brazil is characterized by a low pregnancy rate that has remained stagnant over many years, despite multiple changes in economic and technological factors that affect productivity (IBGE 2020). Low pregnancy rates prevent full development of the livestock sector in many regions of the world, and it may be associated with poor pasture management, overstocking and lack of differential nutritional management for animal categories with different requirements. Stocking rate is considered the most important decision in grazing management because it affects the forage base, herbage allowance, intake and animal performance (Sollenberger, Agouridis, Franzluebbers & Owens 2012). Nutritional limitation during periods of high requirement can compromise development and delay puberty of heifers, as well as inhibit ovulation of cows (Rocha & Lobato 2002).

Time at which puberty occurs relative to the start of breeding season is what determines pregnancy rate in the first breeding season of heifers (Sparke & Lamond 1968), which influences a cow's ability to get pregnant in subsequent years and remain in the herd, determining her lifetime productivity. Puberty of heifers is influenced by management of the annual production cycle, as well as the physiology (production and release of hormones) and its genetic (breed and size of mature age) (Day 2015). In addition to the use of pastures, body weight at the beginning of the breeding season is associated with animal nutrition and it is an important factor influencing the reproductive performance of heifers and beef cows (Richards, Spitzer & Warner 1986; Osoro & Wring 1992; Roso *et al*. 2009).

Natural grasslands and cultivated pastures constitute the forage basis for beef cow herds in many regions of the world, including the US (USDA-ERS 2020). The grasslands that support cow-calf operation in the Pampas are characterized by spring-summer

growth, with quality and availability reduced in autumn and winter. Cultivated and improved pastures are utilized to satisfy the nutritional requirements of cattle, especially during the cooler months when natural pasture growth is limited. Combined with cultivated and improved pastures, supplements may be used to increase average daily gain of grazing animals and to promote greater reproductive development (Frizzo *et al*. 2003).

The aim of the present study was to integrate information from multiple studies of factors that affect pregnancy rates under production conditions in the Pampas to carefully quantify response curves relating pregnancy rate to the most important predictors. First, we take an approach where pregnancy rate is analyzed as a function of known proximate factors such as body weight, category and changes in body weight per day during the breeding season. Second, we add the effects of stocking rate, supplementation and pasture type on proximate factors and directly on pregnancy rate to account for effects not mediated by the proximate factors evaluated. Because of the long term, large geographic region and large number of cows involved in this synthesis, results should be useful not only for ranch-level management but also for regional agricultural policy.

Material and Methods

Ethics Statement

All analyses were based on previously published studies: no ethical approval was required.

Study Sample

Data included records from 29 doctoral dissertations or master's theses for a total of 43 experiments (some studies had more than one experiment). Experiments were selected because the original raw data were available for all of them. Experiments were conducted at the Agronomic Experimental Station of Federal University of Rio Grande do Sul and in private ranches in Rio Grande do Sul, Southern Brazil, to investigate the effects of several factors on pregnancy rate of heifers and primiparous cows (Table 1) between 1976 and 2015. According to Köppen (Wrege, Steinmetz, Reisser Junior & Almeida 2011), climate in all sites represented in the data is subtropical humid.

Table 1 - Relation of studies of database with n (number of animals), location, coordinated geographic, precipitation and type of pasture in southern Brazil

Author	n	Local	Coordinated geographic	Precipitation (mm/year)	Type of pasture*
Albospino, 1990	23	Eldorado do Sul	30º52'/51º39'	1332	Italian ryegrass (*Lolium multiflorum* Lam.)<br>Arrowleaf clover (*Trifolium vesiculosum*)
Azambuja, 2003	216	Arambaré	31º11'/51º74'	-	Natural pasture
Beretta, 1994	113	Eldorado do Sul	30º52'/51º39'	1398	Annual ryegrass (*Lolium multiflorum* Lam.)<br>Arrowleaf clover (*Trifolium vesiculosum*)
Cachapuz, 1976	57	Dom Pedrito	30º99'/54º70'	1376	Natural pasture<br>Common vetch (*Vicia sativa*)
Deresz, 1976	110	Pelotas	30º58'/50º40'	1285	Natural pasture<br>Annual ryegrass (*Lolium multiflorum* Lam.)<br>Arrowleaf clover (*Trifolium vesiculosum*)
Fagundes, 2001	87	Itaqui	29º24'/56º47'	1500	Natural pasture
Freitas, 2005	350	São Gabriel	30º33'/54º32'	1193	Annual ryegrass (*Lolium multiflorum* Lam.) White clover (*Trifolium repens*)<br>Bird's-foot trefoil (*Lotus corniculatus*)
Gottschall, 1994	114	São Gabriel	30º33'/54º32'	1512	Natural pasture
Lopes, 2004	39	Eldorado do Sul	30º52'/51º39'	1446	Black oats (*Avena strigosa*)<br>Annual ryegrass (*Lolium multiflorum* Lam.)<br>Arrowleaf clover (*Trifolium vesiculosum*)<br>Pearl millet (*Pennisetum americanum*)

*Natural pasture with prevalence of Bahiagrass (*Paspalum notatum*), Dallisgrass (*Paspalum dilatatum*), *Axonopus affinis*, *Andropogon lateralis*, *Trifolium polymorphum*

Table 1 ... Continued

Author	n	Local	Coordinated geographic	Precipitation (mm/year)	Type of pasture*
Magalhães, 1992	210	Rosário do Sul	30º25'/54º92'	1550	Annual ryegrass (*Lolium multiflorum* Lam.)
Marques, 2001	231	Eldorado do Sul	30º52'/51º39'	1440	Black oats (*Avena strigosa*)<br>Annual ryegrass (*Lolium multiflorum* Lam.)<br>Arrowleaf clover (*Trifolium vesiculosum*)
Menegaz, 2006	323	Uruguaiana	29º76'/57º09'	1500	Natural pasture<br>Annual ryegrass (*Lolium multiflorum* Lam.) White clover (*Trifolium repens*)<br>Bird's-foot trefoil (*Lotus corniculatus*)
Moraes, 1991	60	Dom Pedrito	30º99'/54º70'	1300	Annual ryegrass (*Lolium multiflorum* Lam.)<br>White clover (*Trifolium repens*)<br>Sorghum (*Sorghum bicolor*)<br>Bird's-foot trefoil (*Lotus corniculatus*)
Müller, 1998	50	Eldorado do Sul	30º52'/51º39'	1440	Annual ryegrass (*Lolium multiflorum* Lam.)<br>Arrowleaf clover (*Trifolium vesiculosum*)
Nardon, 1985	65	Eldorado do Sul	30º52'/51º39'	1398	Annual ryegrass (*Lolium multiflorum* Lam.)<br>Arrowleaf clover (*Trifolium vesiculosum*)
Pereira Neto, 1996	62	Eldorado do Sul	30º52'/51º39'	1332	Annual ryegrass (*Lolium multiflorum* Lam.)<br>Arrowleaf clover (*Trifolium vesiculosum*)
Pilau, 2007	234	Tupanciretã	29º03'/53º48'	-	Black oats (*Avena strigosa*)<br>Annual ryegrass (*Lolium multiflorum* Lam.)
Polli, 1986	71	Eldorado do Sul	30º52'/51º39'	1398	Natural pasture<br>Annual ryegrass (*Lolium multiflorum* Lam.)<br>Arrowleaf clover (*Trifolium vesiculosum*)

*Natural pasture with prevalence of Bahiagrass (*Paspalum notatum*), Dallisgrass (*Paspalum dilatatum*), *Axonopus affinis*, *Andropogon lateralis*, *Trifolium polymorphum*

Table 1... Continued

Author	n	Local	Coordinated geographic	Precipitation (mm/year)	Type of pasture*
Pötter, 2002	92	Quaraí	30º26'/56º01'	1356	Natural pasture<br>Annual ryegrass (*Lolium multiflorum* Lam.)<br>Bird's-foot trefoil (*Lotus corniculatus*)
Quadros, 1991	69	Dom Pedrito	30º99'/54º70'	1540	Natural pasture
Ribeiro, 1986	70	Cachoeira do Sul	30º03'/52º89'	1621	Natural pasture
Rocha, 1997	394	Dom Pedrito	30º44'/54º47'	1450	Annual ryegrass (*Lolium multiflorum* Lam.)<br>White clover (*Trifolium repens*)<br>Red clover (*Trifolium pratensis*)<br>Bird's-foot trefoil (*Lotus corniculatus*)
Rosa, 2010	241	Dom Pedrito	30º44'/54º47'	1300	Natural pasture<br>Annual ryegrass (*Lolium multiflorum* Lam.) White clover (*Trifolium repens*)<br>Bird's-foot trefoil (*Lotus corniculatus*)
Silva, 2010	142	Bagé	31º22'/54º39'	1300	Annual ryegrass (*Lolium multiflorum* Lam.)
Simeone, 1995	119	Bagé	31º22'/54º39'	1350	Natural pasture
Souza, 2005	64	Dom Pedrito	30º99'/54º70'	1376	Annual ryegrass (*Lolium multiflorum* Lam.)<br>White clover (*Trifolium repens* L.)
Souza, 2015	49	Júlio de Castilhos	29º23'/53º68'	-	Black oats (*Avena strigosa* Schreb.)<br>Palisade grass (*Urochloa brizantha)*
Tanure, 2008	194	Quaraí	30º26'/56º01'	1356	Natural pasture
Zanotta Jr, 1984	84	Pelotas	30º58'/50º40'	1285	Natural pasture<br>Annual ryegrass (*Lolium multiflorum* Lam.)<br>White clover (*Trifolium repens*)

*Natural pasture with prevalence of Bahiagrass (*Paspalum notatum*), Dallisgrass (*Paspalum dilatatum*), *Axonopus affinis*, *Andropogon lateralis*, *Trifolium polymorphum*

Data

The initial database created contained the following variables for each of 3933 animals: age at the beginning of the breeding season was 14 to 36 months (average was 24.6±7.5 months); animal categories were heifers (n=2257) and primiparous cows (n=1676); average animal weight was 315.4±55.9 kg at the beginning and 337.3±53.4 kg at the end of the breeding season; body condition score in a 0-5 scale was 3.2±0.6 at the beginning and 3.4±0.6 at the end of the breeding season; breeds were Angus (n=306), Braford (n=499), Brangus (n=323), Crossbred (n=1928), Devon (n=110) and Hereford (n=767); stocking rate ranged from 200 to 464 kg of body weight per hectare (kg BW/ha) and averaged 337.32±54.68 kg BW/ha; pasture types were cultivated (n=2050), improved (n=324) or natural grassland (n=1559); feed supplementation before the breeding season was recorded as a binary variable (supplemented vs. not supplemented).

Breeds were recoded as crossbred (final N = 1924) vs. *B. Taurus* (final N = 1058). One experiment with an extreme stocking rate of 800.0 kg of body weight per hectare was excluded from the analysis. The remaining data had stocking rates ranging from 200 to 463.5 (average was 336.85±54.92) kg BW/ha, which are more typical for the region. Columns with large number of missing values and rows without complete multivariate records were excluded, resulting in a final data set of 2982 records (animals) for which all variables depicted in Figure 1 were available.

Fig. 1. Schematic structure of the hypothesized determinants of pregnancy rate (PR).

Statistical Analyses

Statistical analyses were conducted using R (R Development Core Team 2015). We used generalized linear mixed models (GLMM) based on the logit link function, as they are generally recommended for binary data (Agresti 2002; Sauvant, Schidely, Daudin & St-Pierre 2008). Data for each heifer and primiparous cow were available, allowing an analysis analogous to an incomplete block design, with a random intercept for each experiment (Senn, Gavini, Magrez & Scheen 2011).

The main response variable was pregnancy rate as evaluated by the relation between the number of pregnant heifers or primiparous cows and the total number of heifers or primiparous cows in each experiment. Explanatory factors considered were category, weight at the beginning of the breeding season, weight change during the breeding season, breed, stocking rate, and type of pasture before and after the breeding season. All quantitative variables were standardized to facilitate the convergence of the computations to estimate parameters. A structure of causal effects was established a priori (Figure 1) and then simplified by removing nonsignificant components.

First, pregnancy rate (PR) was analyzed using *glmer* with a binomial distribution and a logit link, as a function of known proximate factors such as body weight and category. Breed was included to account for inherent differences in breeds that could modulate the effects of body weight, for example, due to differences in mature body or frame size. Model selection proceeded by simplification of a full model until it had only those effects that were significant or part of significant interactions. Significance of terms was assessed by type II Wald tests using the Anova() function of the *car* package [27]. The initial full model, expressed as an R formula for the generalized linear mixed-effects model (glmer) function of the lme4 package (Bates, Maechler, Bolker & Walker 2014) was

PR ~ breed * start.bw + categ * s.dwt + s.dwt * breed +

s.dwt * start.bw + I(start.bw ^ 2) + I(s.dwt ^ 2) + (1 | experiment)

where *categ* is animal category, *start.bw* is weight at the beginning of the breeding season, *s.dwt* is daily weight gain, *I(start.bw^2)* is squared weight at the beginning of the breeding season, *I(s.dwt^2)* is daily weight gain squared and *experiment* is a categorical variable or factor with a different value for each experiment. Each experiment was allowed a random effect to account for the potential intraclass correlation caused by common condition for all animals in each experiment. The "*" operator indicates that both main effects and their interaction are included in the model. This final model was tested against the full model by a likelihood-ratio test using the anova() function to make sure they were not significantly different.

Second, stocking rate was added as the last term to the resulting model to determine if stocking rate had significant effects beyond those effected via proximate factors. Significance of stocking rate effects was assessed with the same Wald test as before. Third, body weight at the beginning of the breeding season was modeled with the following full model:

start.bwt ~ categ + breed + pasture.pre * s.sr +

pasture.pre * I(s.sr ^ 2) + sup.pre + (1 | experiment)

where pasture.pre is a factor indicating whether animals grazed natural grassland, cultivated pastures or improved grassland prior to the breeding period; s.sr is stocking rate, and I(s.sr^2) is the quadratic effect of stocking rate. Other terms were defined above. Finally, change in body weight during the breeding period (s.dwt) was analyzed starting with the following full model:

s.dwt ~ categ + breed + pasture.breed + s.sr + sup.breed + I(s.sr^2) + pasture.breed:s.sr + categ:s.sr + breed:s.sr + (1 | experiment)

where pasture.breed is the type of pasture grazed during the breeding period and sup.pre is a binary variable indicating whether animals received supplementation during the breeding period. Models for start.bwt and s.dwt were simplified and final models were tested following the same procedures as before. For all models, assumptions were assessed by inspection of residual plots.

Results

Effects of proximate causal factors on pregnancy rate

The final model for selected pregnancy rate was:

PR ~ categ + start.bw * breed + s.dwt + I(start^2) + (1|experiment).

The most important factor affecting pregnancy rate was body weight at the beginning of the breeding season (Table 2), which accounted for 68% of the model sum of squares. Mc Fadden's pseudo R^2 (Domencich & McFadden 1974) for fixed effects of the complete final model was 9.53%, whereas a model with only the linear and quadratic effects of initial body weight had a pseudo R^2 equal to 8.8%.

Table 2 – Analysis of variance of the model for pregnancy rate with proximate factors.

Effect	Wald's Chi-sq (type II)	df	p-value
start.bw[1]	234.9	1	<0.0001
I(start.bw^2)[2]	48.2	1	<0.0001
s.dwt[3]	11.1	1	0.0009
categ[4]	10.7	1	0.0011
start.bw:breed[5]	6.2	1	0.0141

breed[6]	2.7	1	0.0995

[1]Weight at the beginning of the breeding period (standardized using mean = 318 kg, s = 69 kg).
[2]Quadratic effect of weight at the beginning of the breeding season.
[3]Average daily gain during the breeding season (standardized using mean = 0.264 kg/day, s = 0.314 kg/day).
[4]Animal category (heifer or primiparous cow).
[5]Interaction between weight at the beginning of the breeding season and breed.
[6] Breed type (crossbred or taurine).

Pregnancy rate increased steeply with increasing body weight at the beginning of the breeding season for crossbreed and taurine females, but for taurine females it increased faster and reached a higher maximum than for crossbreed females. Taurine females starting the breeding season with an average weight of 440.0 kg or more and had an expected pregnancy rate of 99.0%, whereas crossbred females that started the breeding season with similar weight had an expected pregnancy rate of 91.0% (Fig 2).

Fig. 2. Interactin between body weight at the beginning of the breeding season and Breed to pregnancy rate of heifers and primiparous cows (Sint: crossbred, Taur: taurine females). Shaded strips represent 95% confidence intervals for the expected value. Body mass average and standard deviation were 318 and 69 kg.

Pregnancy rate increased with increasing average daily gain during the breeding period. Averaging over other predictors, the model estimated that pregnancy rate increases from 59% when animals lose 340 g per day to 79% when they gain 890 g per day during the breeding season. When daily gain was at its average (0.264±0.006 kg per day), pregnancy rate increased 1.7% per 100 g of daily gain during the breeding period. When all covariates are at their average values for both categories, pregnancy rate was 25 percentage points higher for heifers than for primiparous cows (80 vs. 55%).

Effects of stocking rate not mediated by proximate factors

The addition of stocking rate as an explanatory factor resulted in the following model:

preg ~ start.bw + s.dwt + I(start.bw ^ 2) + breed + categ + s.sr + start.bw : breed + s.sr:start.bw + (1 | experiment).

The contribution of stocking rate to explain variation in pregnancy rate was evaluated by entering stocking rate and its interactions last into the model and using a type II Wald test. Stocking rate and its interaction with body weight at the start of the breeding season contributed 4.7% of the total sum of squares of the model and had direct effects on pregnancy rate that were significant even after controlling for the potential indirect effects through proximate variables such as body weight and weight change during breeding (Table 3). Stocking rate exhibited a significant interaction with weight at the start of the breeding season by which the effect of stocking rate was small for body weight below the 318 kg average and negative for heavier animals (Fig 3).

Table 3 - Analysis of variance of the pregnancy rate model with proximate factors and stocking rate

Variables	Wald's Chi-sq (type II)	df	p-value
start.bw	227.4	1	<0.0001
I(start.bw^2)	44.1	1	<0.0001
s.dwt	9.5	1	0.0021
categ	7.7	1	0.0054
start.bw:breed	7.1	1	0.0076
s.sr[1]	6.1	1	0.0135
start.bw:s.sr[2]	6.0	1	0.0144
breed	2.9	1	0.0883

[1]Stocking rate (standardized using mean = 328 kg/ha and s = 58.9 kg/ha).
[2]Interaction between weight at the start of the breeding season and stocking rate.

Fig. 3. Interaction between body weight at the beginning of the breeding season and stocking rate on pregnancy rate of heifers and primiparous cows. Shaded areas are 95% confidence intervals. Tickmarks above the horizontal axis represent observations.

Although stocking rate did have effects on pregnancy rate beyond those through proximate causal variables, the effects of other variables did not change much by the incorporation of stocking rate. The most important factor affecting pregnancy rate when stocking rate was added in the model continued to be body weight at the beginning of the breeding season, accounting for 84.4% of the sum of squares explained by the generalized mixed model (Table 3). The second largest contribution to the sum of squares of the model was due to average daily gain during the breeding season, which accounted for 4.3% of the explained variation (Table 3). The response to daily gain was similar to that in the model without stocking rate; pregnancy rate increased 1.5% per 100 g of daily gain when daily gain was near its average. The third largest contribution to the model's sum of squares was due to animal category, accounting for 3.0% of the explained variation. Pregnancy rate was greater in heifers than in primiparous cows (81 vs. 56%, p = 0.0054).

Body weight at the beginning of the breeding season

Because supplementation did not have detectable effects on body weight at the start of the breeding season, the final model was

start.breed.wt ~ categ + breed + pasture.pre + s.sr + I(s.sr^2) +
pasture.pre:s.sr + pasture.pre:I(s.sr^2) + (1 | experiment),

where *start.breed.wt* is body weight in kg at the start of the breeding season and *pasture.pre* is the type of pasture grazed prior to the breeding season. Thirty nine percent

of the variation in initial body weight was explained by the fixed effects of the model, and an additional 41% was explained by variation among experiments (random effects variation due to differences between experiments in variables not measured). Pasture type accounted for 60% of the sum of squares explained by fixed effects, whereas an additional 27.9% was explained by stocking rate and its interaction with pasture type. Body weight declined quadratically with increasing stocking rate in natural pastures, but it was not affected by the range of stocking rates studied in cultivated or improved pastures (Fig 4). At the average stocking rate of 328 kg/ha, initial body weight of animals grazing cultivated and improved pastures was 15 kg greater than that of animals grazing natural pastures, and this difference increased to 44 kg when stocking rate increased to 388 kg/ha.

Fig. 4. Effects of stocking rate and type of pasture on body weight at the beginning of the breeding season of heifers and primiparous cows. Effects of stocking rate were not significant for cultivated (Pcul) and improved (PNMelh) pastures, but there was a significant quadratic effects in native grasslands (PNat).

Breed type and category had effects on initial weight that were independent of stocking rate. Ninety five percent confidence intervals for weight at the start of the breeding season were 303 to 338 and 295 to 330 kg for crossbred and taurine types. Confidence intervals for heifers and primiparous cows were 263 to 313 and 332 to 380 kg.

This reflects the fact that multiple factors determine pregnancy rate, many of which are not closely related to body weight, animal category or breed. Other factors explored in some experiments, such as weaning method and use of artificial insemination,

were considered and did not have detectable effects in preliminary models for pregnancy rate. However, the different weaning methods and use of artificial insemination were not represented across a good range of values in the other factors.

Change in body weight during the breeding season

The final model for average daily gain during the breeding season was

s.dwt ~ pasture.breed + s.sr + I(s.sr ^ 2) + (1 | experiment),

where *pasture.breed* is the type of pasture grazed during the breeding season.

Stocking rate and type of pasture grazed accounted for equal parts of the total variarion explained by the fixed effects of the model. Average daily gain during the breeding season decreased quadratically with increasing stocking rate (Fig 5).

Fig. 5. Average daily gain during the breeding season and stocking rate of heifers and primiparous cows. Shaded strip shows the 95% confidence band.

Discussion

This study is unique because it integrated data from thousands of individual animals from multiple sites and experiments and because it established quantitative relationships between reproductive performance and stocking rate. Although stocking rate is recognized as one of the most important factors determining productivity of grazing systems (Hunt, Mclvor, Grice & Bray 2014), studies relating grazing animal performance to stocking rate are rare. A Web of Science search performed on 22 July 2021 with the terms “beef catle” AND “pregnancy rate” AND “stocking rate” yielded nine articles, only one of which (Renquist, Oltjen, Sainz, connor & Calvert 2005) presented original data on

the effects of stocking rate on pregnancy rates. Most of the studies where stocking rate is considered as one of the explanatory variables for animal performance include few levels of stocking rate that explore a very limited range. We surmise that as a consequence of the limited range explored and the inherent high variability of herd-level studies, many studies failed to detect effects of stocking rate. The present study included 16 levels of stocking rate ranging from 0.5 to 1.2 head/ha or 200 to 464 kg/ha, which allowed quantification of response curves.

Effects of proximate causal factors on pregnancy rate

The effects of body weight at the beginning of the breeding season, animal category and changes in body weight during the breeding season were quantified and yielded response curves with low variance. For example, the pseudo coefficient of variation (CI half width/(2 expected value) of pregnancy rate for taurine cows at average weight at the beginning of the breeding season was 6.5%. The most important causal factor influencing pregnancy rate was body weight at the beginning of the breeding season, which interacted with breed to determine that at high initial weights, taurine females had higher pregnancy rate than crossbred females (Fig 2). Pregnancy rate is influenced by nutrition, because it directly affects the reproductive physiology in beef cows (Sartori & Guardieiro 2010), mainly in periods of higher requirements like pre and postpartum. If nutrition is inadequate, body reserves become depleted and body condition declines (Diskin & Kenny 2016), resulting in low ovulation rate. Females with adequate metabolic status and high body weight have high levels of glucose, insulin and growth factor I (IGF-I) (Yelich *et al*. 1995; Santos & Amstalden 1998), potentiating the effect of gonadotrophins (LH and FSH) (Spicer & Echternkamp 1995) and promoting ovulation

(Sirois & Fortune 1988). Pre and postpartum periods coincide with low availability of nutrients in natural grasslands, which are characterized by variation in composition, structure and, seasonality of production and quality (Mezzalira *et al*. 2014; Neves *et al*. 2009).

Animals with smaller frame reach physiological maturity earlier, at a lower weight and with greater fat content than larger animals (Owens, Dubeski & Hanson 1993). When growth rate decreases and the process of fat deposition begins, larger animals are still in the growth phase (Mckiernan 2005). In addition, the higher pregnancy rate observed in the taurine females can be explained by the greater selection for precocity carried out in the herds from which these females proceed (Diskin & Kenny 2016). When heifers reach puberty and mate earlier the biological efficiency of the herd is improved for as long as the early mating does not compromise full development. These characteristics become more important as production systems become more intensive and competitive. Reducing the age at first conception alters the structure of the herd and shortens the interval between generations, thus decreasing the participation of unproductive animals in the composition of the herd (Pötter, Lobato & Mielitz Neto 1998; Beretta, Lobato & Mielitz Neto 2001).

Weight gain during the breeding season is clearly important for cows to become pregnant. Greater weight gain in this period indicates that forage is less limiting, and that sufficient quantity and quality of food intake is obtained to support ovarian activity (Vieira, Lobato, Torres Junior, Cezar & Correa 2005). According to Carter & Cox (1973), there is greater biological efficacy in females that have their first calf at about two, rather than three or more years of age. Adequate weights at the beginning of the breeding season are decisive for a high conception rate (Wiltbank, Rowden, Ingalls, Geegoey & Koch 1962).

The lower pregnancy rate of primiparous cows than heifers may be related to the stress of calving and the combined effects of growth and first lactation requirements of primiparous cows. Low reproductive success has been documented for primiparous animals when they are subjected to periods of pre or postpartum feeding restriction (Spitzer, Morrison & Wettemann 1995). The negative direct effect of primiparous condition on pregnancy rate appeared with the inclusion of initial body weight, but primiparous condition had an indirect positive effect on pregnancy rate relative to heifers through the fact that they were heavier than heifers at the beginning of the mating period (Fig 6).

Fig. 6. Schematic representation of modeling resuls showing effects of animal factors (category and breed type), foraging environment (pasture type, stocking rate and supplementation) on body weight and reproductive performance of beef cattle. In the broader lines are observed direct effects on the pregnancy rate. In the thinner lines are observed indirect effects of body weight prior to breeding and in fine and dotted lines, indirect effects of the change in body weight during breeding.

Stocking rate interacted with body weight at the beginning of the breeding season, whereby higher stocking rates were associated with lower pregnancy rate only in the high range of body weight (Fig 3). Lower stocking rates allow greater development of the animal, due to the higher forage accumulation, making it possible for females to have more food available (Euclides & Euclides Filho 1998). Stocking rate is a primary management variable in grazing systems because it modulates the interactions between animals and pasture (Bransby & Maclaurin 2000; Carvalho & Batello 2009). As stocking

rate increases, herbage allowance decreases and can reach levels where intake per animal is too low for production, but intake per unit area surpasses the ability of pastures to produce and recover. Individual animal performance decreases as stocking rate increases, because the daily intake is constrained by limiting sward structure at low herbage allowance (Da Trindade *et al*. 2014), but production per unit area increases and then decreases with increasing stocking rate (Mezzalira *et al*. 2012; Neves *et al*. 2009; Petersen, Lucas & Mott 1965). High stocking rates pre and post partum make it difficult for cows to recover good body condition after calving, compromising the reproductive performance of the cow and the productivity in subsequent seasons and reproductive years (Osoro 1989). Therefore, stocking decisions must be informed by curves that relate individual performance to stocking rate like the ones provided in the present study. These curves are particularly important for the integration of biological and economic functions to determine optimal stocking rates.

As expected, heifers achieve greater pregnancy rates when mantained in good nutritional conditions. Inadequate management practices, such as excessive stocking rate and lack of custom management for certain animal categories have led to generally low indices of productivity in the region. However, there are possibilities for reducing the age of slaughter and the age at first breeding, which may allow the improvement of productive and reproductive indices (Pötter, Lobato & Mielitz Neto 2000; Beretta, Lobato & Mielitz Neto 2002). Because natural pastures of the region are dominated by warm season grasses with low productivity and quality in the cold season, grazing of cultivated cool-season pastures significantly increases indicators of reproductive performance, beef yield and economic results (Lupatini & Neumann 2002).

In agreement with (Rovira 1974), we observed that when heifers and primiparous cows are mantained in optimal conditions of grazing and nutrition, that is, mantained in high quality pastures, with sufficient body weight and intermediate stocking rates, they achieve near maximal reproductive success. Sufficient nutrition allows early breeding, which increases the overall efficiency of production for the herd.

Effects of stocking rate not mediated by proximate factors

Our results show that stocking rate has an effect on pregnancy rate that is not explained by any of the other variables considered. Even after controlling for effects of starting body weight and weight change during the breeding season, when body weight at the beginning of the breeding season is greater than average, pregnancy rate declines with increasing stocking rate. This effect of stocking rate appears to be restricted to the range of starting body weight where pregnancy rate no longer responds to body weight. This further suggests that stocking rate had an effect that was not mediated by the observed effects of stocking rate on body weigh at the start of the breeding period and weight change during breeding (Fig 6).

Effects of stocking rate on pregnancy rate that are not related to nutritional condition, as reflected in body weight and daily gain, might be related to animal health and associated management variables. Higher stocking rates may result in greater load of external and internal parasites (Gasser 2013). Tick infestation is common in this region, and ticks frequently carry Babesia (Bilhasi *et al*. 2014). When grazing at higher stocking rates, animals are forced to graze closer to the soil and increase the rate of ingestion of parasite helminth larvae.

Body weight at the start of breeding season and changes in body weight during the breeding season

Our results agree with the conventional wisdom that stocking rate is one of the most important factors in grazing management. Stocking rate interacting with type of pasture before mating was the most important factor influencing body weight at the beginning of the breeding season (Fig 4) and it was the most important factor affecting the changes in body weight during the breeding season (Fig 5). The lowest weights at the beginning of the breeding season observed in this study are close to the minimum weight recommended for the first breeding season (50-57% of adult weight) to avoid impairment of life-long reproductive performance (Funston & Deutscher 2004; Roberts, Geary, Grings, Waterman & MacNeil 2009). Body weight of heifers and cows grazing cultivated or improved pastures was high and did not respond to stocking rate, presumably because the level of feeding and pasture production was sufficient to provide enough nutrition at all stocking rates studies. On the other hand, we observed a typical response of declining in body weight and average daily gain during the breeding season for animals grazing native pastures where forage amount and quality are lower than in cultivated and improved pastures. Lower stocking rates allow animals to select better-quality diets, while higher stocking rates reduce vegetation abundance, constraining daily intake (Da Trindade *et al*. 2016).

Conclusions

This synthesis of a large number of experiments conducted over decades in the Pampas region confirms the importance of body weight at the start of the breeding season

to achieve high pregnancy rates in cattle. Body weight at the beginning of the breeding season is an easily measurable variable and can be used herd reproductive management. Stocking rate had a negative effect on pregnancy rate both through its negative effects on initial body weight and weight change during the breeding season, and its direct negative effects on pregnancy rates when body weight was not limiting. Heifers tended to have lower body weight than primiparous cows, but after correction for body weight, they had greater pregnancy rates than primiparous cows, most likely due to the fact that primiparous cows were simultaneously lactating and growing. Response curves derived from out study can be used to optimize stocking rates under various economic conditions and to guide policies to improve the efficiency of reproductive livestock herds under free-grazing conditions.

References

Agresti A. Categorical data analysis, 2nd edition. John Wiley & Sons, Hoboken. New Jersey; 2002.

Bates D, Maechler M, Bolker B, Walker S. Lme4: linear mixed effects models using Eigen and S4.R package version 1.1-11. Available at http://CRAN.R-project.org/package=lme4.

Beretta V, Lobato JFP, Mielitz Neto CGA. Productivity and Biological Efficiency of Beef Cow-Calf Systems Differing in Age at First Calving and Herd Calving Rate in Rio Grande do Sul. Braz J Anim Sci. 2001; 30: 1278-1286.

Beretta V, Lobato JFP, Mielitz Netto CGA. 2002. Productivity and biological efficiency of alternative beef cattle life-cycle production systems in Rio Grande do Sul. Braz J Anim Sci. 2002; 31: 991-1001.

Bilhassi TB, Oliveira HN, Ibelli AMG, Giglioti R, Regitano LCA, Oliveira-Sequeira TCG, Bressani FA, Malagó W, Resende FD, Oliveira MCS. Quantitative study of Babesia bovis infection in beef cattle from São Paulo state, Brazil. Ticks and Tick-Borne Diseases. 2004; 5: 234-238.

Bransby DI, Maclaurin AR. Designing animal production studies. In: T'Mannetje, L., R. M. Jones, editor, Field and laboratory methods for grassland and animals production research. CAB International, Wallingford; 2000; pp.327-352.

Carter AH, Cox EH. Observations on yearling mating of beef cattle. In: New Zealand Society of Animal Production. Proceedings. Palmerston North; 1973; pp.94-113.

Carvalho PCF, Batello C. Acess to land, livestock production and ecosystem conservation in the Brazilian Campos biome: The natural grasslands dilemma. Livestock Science. 2009; 120: 158-162.

Da Trindade JK, Neves FP, Pinto CE, Bremm C, Mezzalira JC, Nadin LB, Genro TCM, Gonda HL, Carvalho PCF. Daily forage intake by cattle on natural grassland: response to forage allowance and sward structure. Rangeland Ecology & Management. 2016; 69: 59-67.

Day ML. Nutritional effects on beef heifer development, puberty and subsequent reproduction. Proc. Florida Ruminant Nutrition Symposium. 2015; 26: 69-77.

Diskin MG, Kenny DA. Managing the reproductive performance of beef cows. Theriogenology. 2016; 86: 379-387.

Domencich T, McFadden D. Urban travel demand: A behavioral analysis; 1974 [cited 2021 July 22]. Available from: http://eml.berkeley.edu/~mcfadden/travel.html.

Duffield TF, Merrill JK, Bagg RN. Meta-analysis of the effects of monensin in beef cattle on feed efficiency, body weight gain, and dry matter intake. Journal of Animal Science. 2012; 90: 4583–4592.

Euclides VPB, Euclides Filho K. Uso de animais na avaliação de forrageiras. EMBRAPA-CNPGC; 1998.

Fagundes JIB, Lobato JFP, Schenkel FS. Effect of two stocking rates on natural pasture and two weaning ages on primiparous cows performance. Braz J Anim Sci. 2003; 32: 1722-1731.

Fisher DS. Defining the experimental unit in grazing trial. In: American Society of Animal Science. Proceedings. Watkinsville; 1999; pp.1-5.

Fox J, Weisberg S. An {R} Companion to Applied Regression, Second Edition. Thousand Oaks CA: Sage. 2011. URL: http://socserv.socsci.mcmaster.ca/jfox/Books/Companion.

Frizzo A, Rocha MG, Restle J, Montagner DB, Freitas FK, Santos DT. Energy supplementation for rearing of beef heifers kept on cool season pasture. Braz J Anim Sci. 2003; 32: 643-652.

Funston RN, Deutscher GH. Comparison of target breeding weight and breeding date for replacement beef heifers and effects on subsequent reproduction and calf performance. Journal of Animal Science. 2004; 82: 3094-3099.

Gasser CL. Joint Alpharma-Beef Species Symposium: Considerations on puberty in replacement beef heifers. Journal of Animal Science. 2013; 91: 1336-1340.

Gottschall CS, Lobato JFP. Comportamento reprodutivo de vacas de corte primíparas submetidas a três lotações em campo nativo. Revista Brasileira de Zootecnia. 1996; 25: 46-57.

Hunt LP, Mclvor JG, Grice AC, Bray SG. Principles and guidelines for managing cattle grazing in the grazing lands of northern Australia: stocking rates, pasture resting, prescribed fire, paddock size and water points – a review. The Rangeland Journal. 2014; 36: 105-119.

ibge.gov.br [Internet]. IBGE – Instituto Brasileiro de Geografia e Estatística; 2020 [cited 2021 Aug 22]. Available from: <http:// http://www.ibge.gov.br/home/estatistica/economia/agropecuaria/indicadoresagro_19962003/default.shtm>.

Lean IJ, Thompson JM, Dunshea FR. A meta-analysis of Zilpaterol and Ractopamine effects on feedlot performance, carcass trail and shear strenght of meat in cattle. PloSv one. 2014; 12: 1-28.

Lovatto PA, Lehnen CR, Andretta I, Carvalho AD, Hauschild L. Meta-analysis in scientific research: a methodological approach. Braz J Anim Sci. 2007; 36: 285-294.

Lupatini G, Neumann M. Planejamento forrageiro para bovinos de corte. In: Encontro de Integração Lavoura-Pecuária no Sul do Brasil. Anais, Pato Branco; 2002; pp.189-216.

Mckiernan B. Frame scoring of beef cattle; 2005 [cited 2021 July 22]. Available from: <http://dpi.nsw.gov.au/animals-and-livestock/beef-cattle/appraisal/publications2/frame-scorign>.

Mezzalira JC, Carvalho PCF, Trindade JK, Bremm C, Fonseca L, Amaral MF, Reffatti MV. Animal and vegetal production of a natural pasture under diferente forage allowances for cattle. Cienc Rural. 2012; 42: 1264-1270.

Neves FP, Carvalho PCF, Nabinger C, Jacques AVA, Carassai IJ, Tentardini F. Herbage allowance management strategies to raise beef heifers on natural pastures. Braz J Anim Sci. 2009; 38: 1532-1542.

Osoro K. Manejo de las reservas corporales y utilización del pasto en los sistemas de producción de carne con vacas madres establecidos en zonas húmedas. Investigación Agraria: Producción y Sanidad Animales. 1989; 4: 207-240.

Osoro K, Wright IA. Pregnancy rate in the first breeding season of heifers is determined primarily by time at which puberty occurs relative to start of breeding season. Journal of Animal Science. 1992; 70: 1661-1666.

Owens FN, Dubeski P, Hanson CF. Factors that alter the growth and development of ruminants. Journal of Animal Science. 1993; 71: 3138-3150.

Petersen RG, Lucas HL, Mott GO. Relationship between rate of stocking and per animal per acre performance on pasture. Agronomy Journal. 1965; 57: 27-30.

Pötter L, Lobato JFP, Mielitz Netto CGA. Productivity of a model of production for primiparous beef heifers at two, three and four years of age. Braz J Anim Sci. 1998; 27: 613-619.

Pötter L, Lobato JFP, Mielitz Netto CGA. Economic analyses of a production model to primiparous beef heifers at two, three and four years of age. Braz J Anim Sci. 2000; 29: 861-870.

Pötter L, Rocha MG, Roso D, Costa VG, Glienke CL, Rosa ATN. Concentrate supplementation for beef heifers on cool-season cultivated pastures. Braz J Anim. Sci. 2010; 39: 992-1001.

Quadros SAF, Lobato JFP. Efeitos da lotação no comportamento reprodutivo de vacas de corte primíparas. Rev Bras Zoot. 1996; 25: 22-35.

r-project.org [Internet]. R: A Language and Environment for Statistical Computing; 2021. [cited 2021 July 22]. Available from: http://www.R-project.org/.

Renquist BJ, Oltjen JW, Sainz RD, Connor JM, Calvert CC. Effects of supplementation and stocking rate on body condition and production parameters of multiparous beef cows. Animal Science. 2005; 81: 403–411.

Richards MW, Spitzer JC, Warner MB. Effect of varying levels of postpartum nutrition and body condition at calving on subsequent reproductive performance in beef cattle. Journal of Animal Science. 1986; 62: 300-306.

Roberts AJ, Geary TW, Grings EE, Waterman RC, MacNeil MD. Reproductive performance of heifers offered ad libitum or restricted access to feed for a one hundred forty-day period after weaning. Journal of Animal Science. 2009; 87: 3042-3052.

Rocha MG, Lobato JFP. Post weaning feeding systems for beef heifers to be bred at 14/15 months of age**.** Braz J Anim Sci. 2002; 31: 1814-1822.

Roso D, Rocha MG, Pötter L, Glienke CL, Costa VG, Ilha GF. Alternatives of utilization of Italian ryegrass pasture for rearing of beef heifers. Braz J Anim Sci. 2009; 38: 240-248.

Rovira J. Reproduccion y manejo de los rodeos de cria. Montevideo: Hemisferio Sur; 1974. 293p.

Santos JEP, Amstalden M. Effects of nutrition on bovine reproduction. Arq. Fac. Vet. UFRGS. 1998; 26: 19-89.

Sartori R, Guardieiro MM. Nutritional factors associated with reproduction in heifers and cows. Braz J Anim Sci. 2010; 39: 422-432.

Sauvant D, Schmidely P, Daudin JJ, St-Pierre NR. Meta-analyses of experimental data in animal nutrition. Animal. 2008; 8: 1203-1214.

Schwarzer G, Carpenter JR, Rücker G. Meta-Analysis with R. In: network Meta-Analysis. Springer, 2015. p.190.

Senn S, Gavini F, Magrez D, Scheen A. Issues in performing a network meta-analysis. Statistical Methods in Medical Research. 2011; 2: 169-189.

Simeone A, Lobato JFP. Efeitos da lotação animal em campo nativo e do controle da amamentação no comportamento reprodutivo de vacas de corte primíparas. Rev Bras Zoot. 1996; 25: 1216-1227.

Sirois J, Fortune JE. Ovarian follicular dynamics during the estrous cycle in heifers monitored by real-time ultrasonography. Biology of Reproduction. 1988; 39: 308-317.

Sollenberger LE, Agouridis CT, Vanzant ES, Franzluebbers AJ, Owens LB. Prescribed grazing on pasturelands. In: C. J. Nelson, editor, Conservation outcomes from pastureland and hayland practices: Assessment, recommendations, and knowledge gaps. Allen Press, Lawrence, KS. 2012; p. 111-204.

Sparke EJ, Lamond DR. The influence of supplementary feeding on growth and fertility of beef heifers grazing natural pastures. Australian Journal of Experimental Agriculture and Animal Husbandry. 1968; 8: 425-433.

Spicer LJ, Echternkamp SE. The ovarian insulin and insulin-like growth factor system with an emphasis on domestic animals. Domestic Animal Endocrinology. 1995; 12: 223-245.

Spitzer JC, Morrison DG, Wettemann RP. Reproductive responses and calf birth and weaning weights as affected by body condition at parturition and postpartum weight gain in primiparous beef cows. Journal of Animal Science. 1995; 73: 1251-1257.

Ers.usda.gov [Internet]. Cattle and Beef. Background, 2020 [cited 2021 July 22]. Available from: https://www.ers.usda.gov/topics/animal-products/cattle-beef/background/.

Vieira A, Lobato JFP, Torres Junior RDS, Cezar IM, Correa ES. Factors affecting the reproductive performance of Nellore cows on the Cerrado conditions of Central Brazil. Braz J Anim Sci. 2005; 34: 2408-2416.

Wiltbank JN, Rowden WW, Ingalls JE, Geegoey KE, Koch RM. Effect of energy level on reproductive phenomena of mature Hereford cows. Journal of Animal Science. 1962; 21: 219-225.

Wrege MS, Steinmetz S, Reisser Júnior C, Almeida IR. Atlas climático da Região Sul do Brasil: Estados do Paraná, Santa Catarina e Rio Grande do Sul. 1. Embrapa Clima Temperado; Colombo: Embrapa Florestas, 2011. 336p.

Yelich JV, Wettemann RP, Dolezal HG, Lusby KS, Bishop DK, Spicer LJ. Effects of growth rate on carcass composition and lipid partitioning at puberty and growth hormone, insulin-like growth factor I, insulin, and metabolites before puberty in beef heifers. Journal of Animal Science. 1995; 73: 2390-2405.

Click here to access/download;Figure;Figure 1.docx

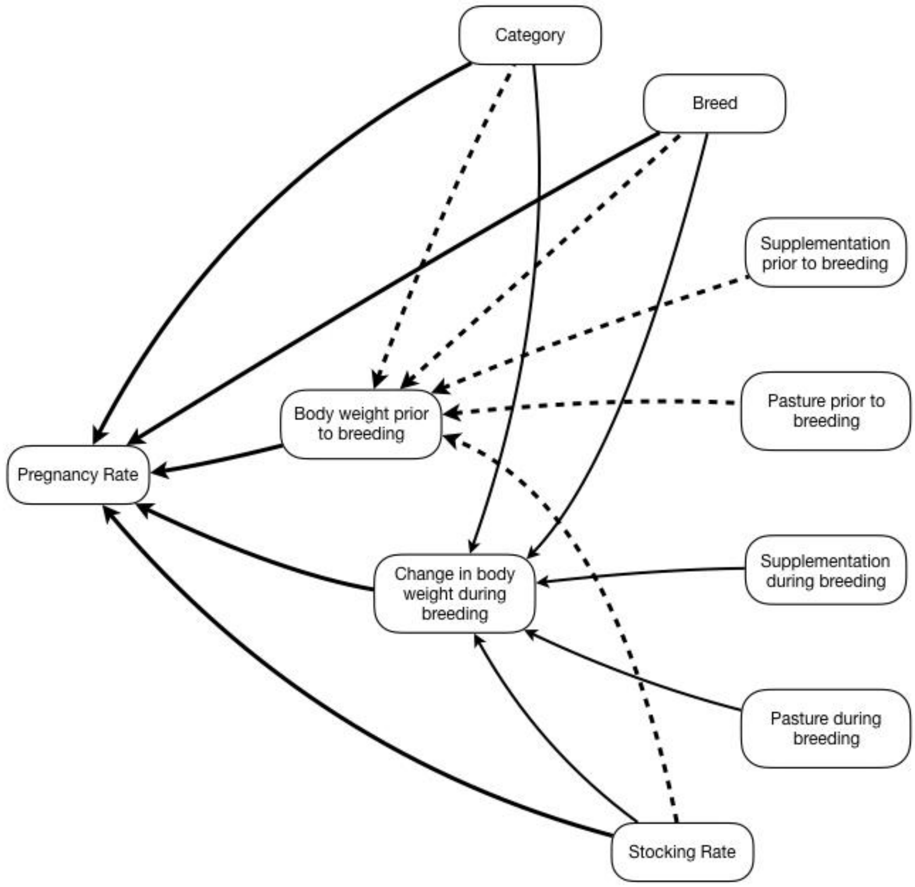

Figure 1. Schematic structure of the hypothesized determinants of pregnancy rate (PR).

 Click here to access/download;Figure;Figure 2.docx

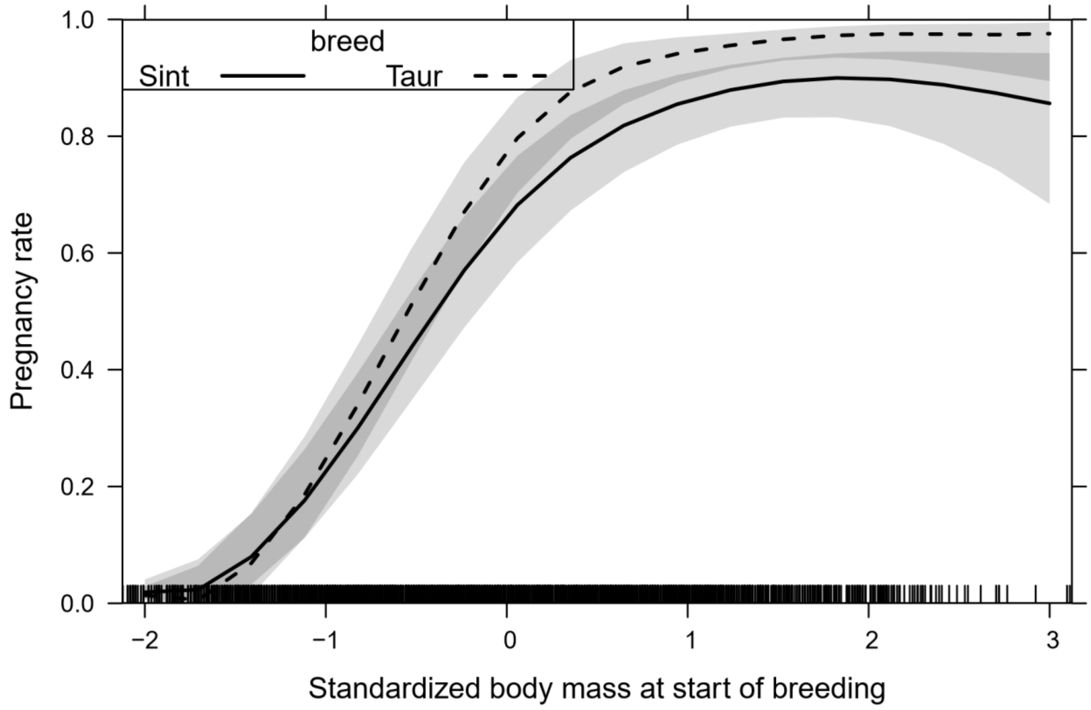

Figure 2. Interaction between body weight at the beginning of the breeding season and Breed to pregnancy rate of heifers and primiparous cows (Sint: crossbred, Taur: taurine females). Shaded strips represent 95% confidence intervals for the expected value. Body mass average and standard deviation were 318 and 69 kg.

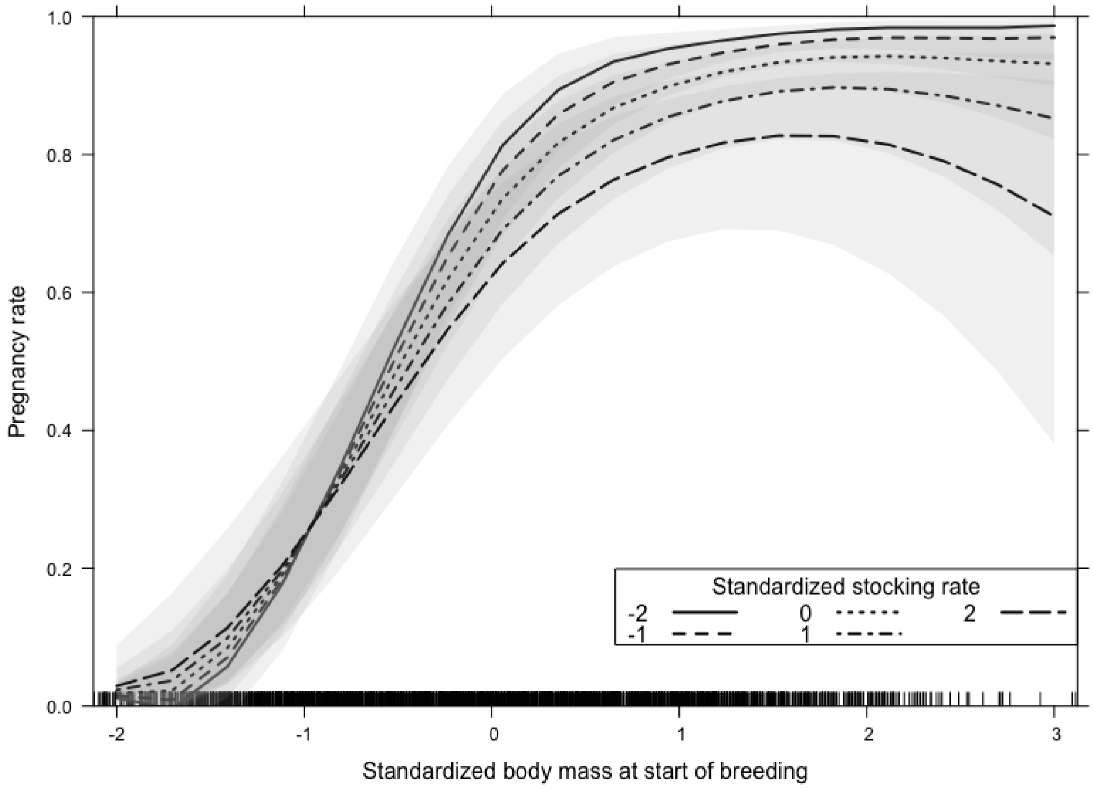

Figure 3 – Interaction between body weight at the beginning of the breeding season and stocking rate on pregnancy rate of heifers and primiparous cows. Shaded areas are 95% confidence intervals. Tickmarks above the horzintal axis represent observations.

Figure 4 Click here to access/download;Figure;Figure 4.docx

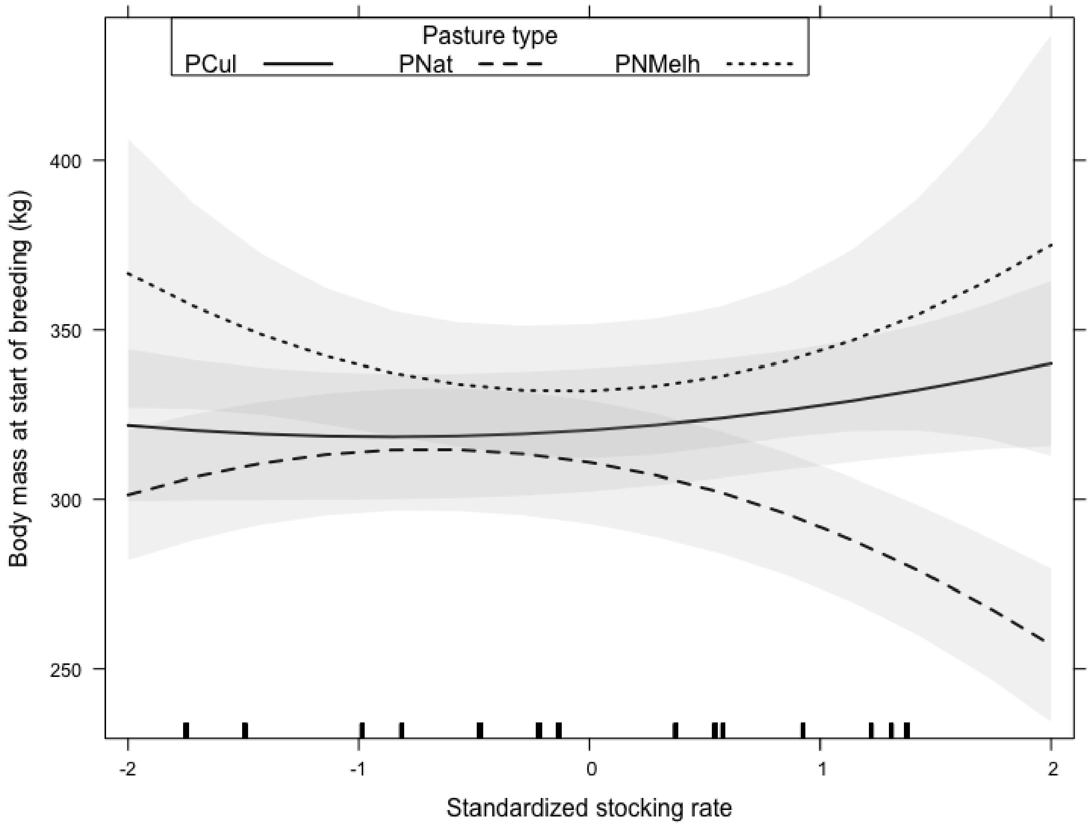

Figure 4 – Effects of stocking rate and type of pasture on body weight at the beginning of the breeding season of heifers and primiparous cows. Efects of stocking rate were not significant for cultivated (PCult) and improved (PNMelh) pastures, but there was a significant quadratic effect in native grasslands (PNat).

Figure 5

Click here to access/download;Figure;Figure 5.docx

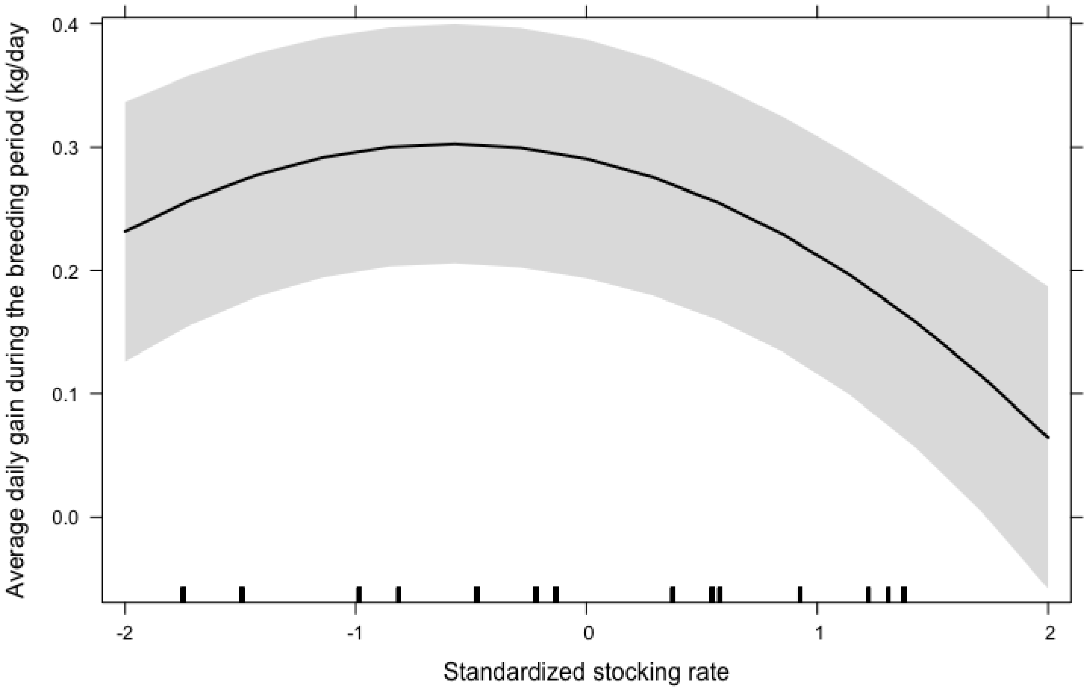

Figure 5 – Average daily gain during the breeding season and stocking rate of heifers and primiparous cows. Shaded strip shows the 95% confidence band.

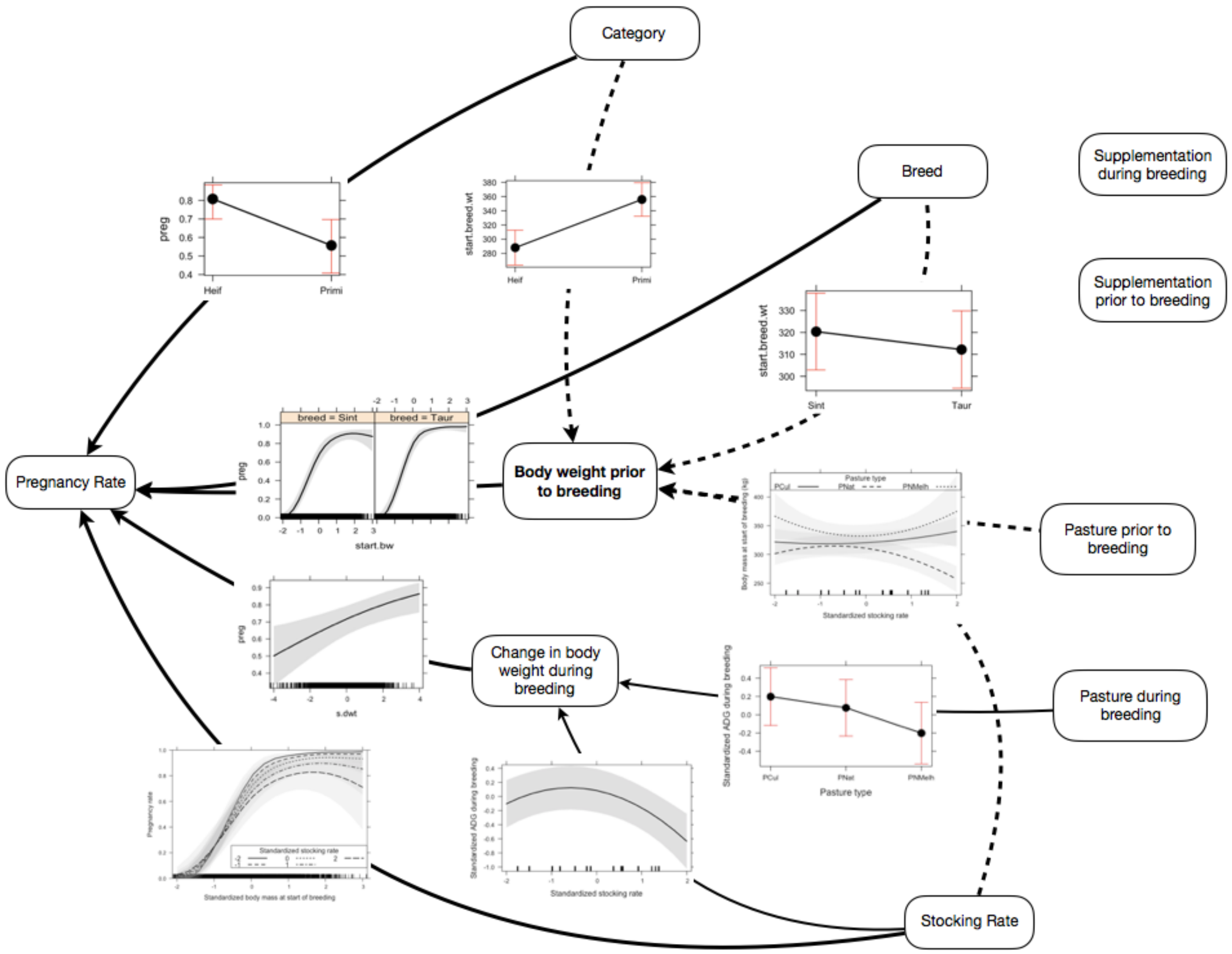

Figure 6. Schematic representation of modeling results showing effects of animal factors (category and breed type), foraging environment (pasture type, stocking rate and supplementation) on body weight and reproductive performance of beef cattle. In the broader lines are observed direct effects on the pregnancy rate. In the thinner lines are observed indirect effects of body weight prior to breeding and in fine and dotted lines, indirect effects of the change in body weight during breeding.
