# Supplementary material for: Direct and indirect nutritional factors that determine reproductive performance of heifer and primiparous cows
Source: PLoS One. 2022 Oct 4;17(10):e0275426. doi: 10.1371/journal.pone.0275426 (PMC9531839; doi:10.1371/journal.pone.0275426)
Supplement: Attachment — Submitted filename: Response to Reviewers PlosOne.doc [file pone.0275426.s003.doc]

PONE-D-21-30153

Direct and indirect nutritional factors that determine reproductive performance of heifer and primiparous cows

PLOS ONE

Dear Academic Editor,

The authors appreciate the comments of the editor and the reviewers, and have accepted the suggestions, which contributed to the improvement of the manuscript.

Changes were made using 'track changes' in MS Word document. Please find below the specific answers to the editor and reviewers’ comments.

**Responses to the Editor’s comments**

Ethics Statement: “N/A”

Editor comment: Shouldn’t it include some statement related to the data collected directly from farmers?

Response: Our study did not involve any data collected directly from farmers. We compiled data and descriptions of studies from published articles, theses and dissertations.

Cover Letter: Most of these experiments were never published before.

Editor comment: Later in the document they say that these experiments were already published elsewhere.

Response: We compiled data and descriptions of studies from published articles, theses and dissertations. The cover letter should have stated that “Most of these experiments were never published in journal articles before.

Introduction:

L62-69: An alternative to specific comprehensive studies is to analyze data pooled form multiples studies (Duffield, Merril & Bagg 2012; Lean, Thompson & Dunshea 2014) that address the same research question using equivalent response and explanatory variables, that is, a meta-analysis (Schwarzer, Carpenter & Rücker 2015). Meta-analysis requires care in the process of systematizing results from multiple studies but it has the advantage of increasing precision, decreasing costs and research time, and increasing the degrees of freedom in the analysis (Fisher 1999; Lovatto, Lehnen, Andretta, Carvalho & Hauschild 2007).

Editor comment: I would suggest not to use the term meta-analysis in the introduction as it can be confusing for the reader and the data used in this manuscript have not been published.

Response: Instead of referring to meta-analysis we now refer to the study as a joint analysis of multiple experiments.

Material and Methods:

L113-114 - Ethics Statement: All analyses were based on previously published studies: no ethical approval was required.

Editor comment: This is confusing. The cover letter indicates otherwise.

Response: We only used published data. The confusion was caused because we were not clear about where the data had been published. Most of the data and experiments were reported and published in theses and dissertations.

Data:

L133-143: The initial database created contained the following variables for each of 3933 animals: age at the beginning of the breeding season was 14 to 36 months (average was 24.6±7.5 months); animal categories were heifers (n=2257) and primiparous cows (n=1676); average animal weight was 315.4±55.9 kg at the beginning and 337.3±53.4 kg at the end of the breeding season; body condition score in a 0-5 scale was 3.2±0.6 at the beginning and 3.4±0.6 at the end of the breeding season; breeds were Angus (n=306), Braford (n=499), Brangus (n=323), Crossbred (n=1928), Devon (n=110) and Hereford (n=767); stocking rate ranged from 200 to 464 kg of body weight per hectare (kg BW/ha) and averaged 337.32±54.68 kg BW/ha; pasture types were cultivated (n=2050), improved (n=324) or natural grassland (n=1559); feed supplementation before the breeding season was recorded as a binary variable (supplemented vs. not supplemented).

Editor comment: Very long paragraph. This could be a summary table.

Response: The authors made a table with the information.

Table 2 – Average and standard error of each variable in the initial database

| Variable | Average |
| --- | --- |
| Age at the beginning of the breeding season^1^ | 24.6±7.5 months |
| Animal categories  Heifers  Primiparous cows | 2257 females  1676 females |
| Body weigth at the beginning of the breeding season | 315.4±55.9 kg |
| Body weigth at the end of the breeding season | 337.3±53.4 kg |
| Breeds  Angus  Braford  Brangus  Crossbred  Devon  Hereford | 306 females  499 females  323 females  1928 females  110 females  767 females |
| Body condition score at the beginning of the breeding season^2^ | 3.2±0.6 |
| Body condition score at the end of the breeding season^2^ | 3.4±0.6 |
| Stocking rate^3^ | 337.32±54.68 kg BW/ha |
| Pasture types  Cultivated  Improved pasture^4^  Natural grassland | 2050  324  1559 |
| Feed supplementation before the breeding season  Not supplemented  Supplemented  Brown rice bran  Commercial concentrate  Corn grain  Deffated rice bran  Deffated rice bran and sorgum silage  Ground corn grain  Sorghum silage and commercial concentrate  Protein salt  Rice and soy bran  Ryegrass and White clover hay  Sectaria hay  Sorghum silage and commercial concentrate | -  0.5 to 1.0% BW  0.7 to 1.5% BW  0.5% BW  1.5% BW  1.5% BW  0.7% BW  1.5% BW  0.1% BW  0.56% BW  0.28% BW  0.92% BW  1.5% BW |

^1^range 14 to 36 months; ^2^0 to 5 scale; ^3^range 200 to 464 kg of body weight per hectare; ^4^Improved pasture were natural pastures with addition of fertilizer and seed of cultivated species in broadcast or sod-seeding applications.

Results:

L237: **Fig. 2.** Interactin between body weight at the beginning of the breeding season and Breed 238 to pregnancy rate of heifers and primiparous cows (Sint: crossbred, Taur: taurine 239 females). Shaded strips represent 95% confidence intervals for the expected value. Body 240 mass average and standard deviation were 318 and 69 kg.

The phrase was re-written as follows:

Fig. 2. Interaction between body weight at the beginning of the breeding season and Breed on pregnancy rate of heifers and primiparous cows (Sint: crossbred, Taur: taurine 2females). Shaded strips represent 95% confidence intervals for the expected value. Body 240 mass average and standard deviation were 318 and 69 kg.

Results:

L295-297: Pasture type accounted for 60% of the sum of squares explained by fixed effects, whereas an additional 27.9% was explained by stocking rate and its interaction with pasture type.

Editor comment: Is this still for initial body weight? It’s confusing that you just mentioned 39% due to fixed effects and 41% by variation among experiments.

Response: Yes, this is still for initial body weight. Variation explained by model = 80% = 39% due to fixed effects + 41% due to random effects. Of the 39% explained by fixed effects, 0.6*39% is explained by pasture type and 0.279 * 39% was explained by pasture type x stocking rate interaction.

We changed the text as follows:

Pasture type accounted for 60% of the sum of squares explained by fixed effects, that is, 0.6 * 39% = 23.4% of the total variation in initial body weight. The interaction between pasture type and stocking rate accounted for 27.9% of the sum of squares explained by fixed effects, that is, 0.279 * 39% = 10.9% of the total variation in initial body weight.

Results:

L304-307: **Fig. 4.** Effects of stocking rate and type of pasture on body weight at the beginning of the breeding season of heifers and primiparous cows. Effects of stocking rate were not significant for cultivated (Pcul) and improved (PNMelh) pastures, but there was a significant quadratic effects in native grasslands (PNat).

Editor comment: This is a result that should not be written in the figure caption.

Response:

The text was removed from the caption.

Figure 4. Pag. 43: **Fig. 4.** Effects of stocking rate and type of pasture on body weight at the beginning of the breeding season of heifers and primiparous cows. Effects of stocking rate were not significant for cultivated (Pcul) and improved (PNMelh) pastures, but there was a significant quadratic effects in native grasslands (PNat).

Editor comment: This is a result that should not be written in the figure caption. Also, include what PCul, PNat and PNMelh mean.

Response: The text was changed to:

**Fig. 4.** Effects of stocking rate and type of pasture on body weight at the beginning of the breeding season of heifers and primiparous cows. Pcul: cultivated pastures; PNMelh: improved pastures; PNat: native grasslands.

**Responses to the Reviewer 1 comments**

Abstract: In the 2^nd^ to last sentence, the phrase “…as well as allows higher in stocking rate…” substitute “an increase” for “higher”.

Response: According to suggestions made by the reviewer, the word “higher” was replaced with “an increase ".

L100-108: The authors do a good job of outlining the aim (objective) of the study, along with the approach to investigate their aim. However, no direct hypothesis is stated. I suggest the authors include a specific hypothesis in this section, and refer to and substantiate this hypothesis in the conclusion of the manuscript.

Response: we added the following hypothesis:

Our main hypothesis is that increases stocking rate will lead to reduced pregnancy rate, and that stocking rate effects on pregnancy rate are mediated by effects of stocking rate on body weight through weight gain during mating.

In the conclusion we added:

The negative effect of stocking rate through body weight and weight gain supports our hypothesis that increases in stocking rate will lead to reduced pregnancy rate, and that stocking rate effects on pregnancy rate are mediated by body weight and weight gain. However, the presence of a direct effect of stocking rate not mediated by body weight or weight gain is strong evidence against the hypothesis that all effects were mediated by body weight. The mechanisms by which stocking rate affects pregnancy rate independently of body weight need further elucidation.

Table 1: This is an extensive and useful table to fully demonstrate the extent and potential application of the results across a wide expanse of conditions.

Response: We agree that the study provides information with wide applicability, particularly in conditions like southern Brazil.

L141: Provide a brief, yet informative statement to better define the term "improved".

Response: We added the following sentence:

Improved pastures were natural pastures with addition of fertilizer and seed of cultivated species in broadcast or sod-seeding applications.

L143: Provide greater detail concerning the type, level and range of supplementation used.

Response: We added type, level and range of supplementation used in Table 2.

L231-232: The result of taurine (straight-bred) pregnancy rate increasing faster and higher maximum than crossbreed females is inconsistent with numerous evidence showing the impact of hybrid vigor on reproductive traits. Authors should address this finding. This result is also noted in lines 356-357, and referenced in Figure 2. In line 373, selection for precocity is a cause for this result, however this appears to be conjecture without evidence.

Response: We added the following sentence and the corresponding citation and reference:

The higher pregnancy rate observed in *B. taurus* cows may be due to the higher selection experienced by these females or to the smaller size and lower milk production of those relative to crossbreed cows, which have higher weight and milk production (Cundiff, 2006).

L450: "weigh" should be corrected to "weight".

Response: According to suggestions made by the reviewer, the word “weigh” was corrected to “weight".

L452-458: Could the decrease in pregnancy rate also be attributed to simply the stress of increased crowding and grazing competition of the animals at higher stocking rates?

Response: Yes, although we think that most of the grazing competition effect would be reflected in weight gain and body weight. We added the following sentence in the discussion:

We surmise that direct effects of stocking rate on pregnancy rate could also be attributed to crowding and other social interactions that impact well-being, as well as competition among bulls.

L484: suggest "use as a reproductive management tool".

Response: The sentence was changed to:

Body weight at the beginning of the breeding season is an easily measurable variable and can be used as a herd reproductive management tool.

L491: correct "out" to "our".

Response: According to suggestions made by the reviewer, the word “out” was corrected to “our".

L656: Gregory is misspelled.

Response: The correct last name is “Geegoey”. It’s corrected in the line 656.

Fig. 3 - horizontal is misspelled.

Response: The word “horzintal” was corrected to “horizontal”.

Fig. 4 - is it possible to include the level of significance (i.e. P value) directly the figure?

Response: Yes. P-values were added to the figure and an explanation was added to the caption, which now reads:

**Fig. 4.** Effects of stocking rate and type of pasture on body weight at the beginning of the breeding season of heifers and primiparous cows. Pcul: cultivated pastures; PNMelh: improved pastures; PNat: native grasslands. P-values refer to the hypotheses that stocking rate has no effect on body weight at the start of the breeding season. Tick marks immediately above the X axis indicate the values of stocking rate present in the sample.

Fig. 5 - similar to comment above, include evidence of quadratic decrease as stated in line 327 with the P value or similar notation directly in the figure. Also, suggest describing what the tick marks on the X axis are.

Response: This comment is addressed above. P-values and explanation were added.

Fig. 6 - An illustrative representation of factors influencing pregnancy rate. I suggest that more visual contrast in the thickness of the lines (thickness) would improve the clarity of the diagram.

Response: The figure was reformatted to be clearer. Figure 1 was also reformatted to be consistent with Figure 6. The new caption is now:

Figure 6. Schematic representation of modeling results showing effects of animal factors (category and breed type), foraging environment (pasture type, stocking rate and supplementation) on body weight and reproductive performance of beef cattle. Full lines represent direct effects, dotted lines represent indirect effects through change in body weight during breeding, and dashed lines represent indirect effects through body weight prior to breeding. Absence of a line indicates the factor was not included in the final model.

**Responses to the Reviewer 3 comments**

L231 the phrase 'taurine females' is used and this is new to me, I have never seen this before. If I had to guess it means 'purebred.' but either a definition should be given or please use a different word.

Response: We have replaced the word “taurine” with “*B. taurus*” throughout.

L339 cattle is misspelled.

Response: According to suggestions made by the reviewer, the word “catle” was corrected to “cattle”.

L 481, perhaps the word 'analysis' is more correct than 'synthesis' in this context.

Response: The sentence was modified and now reads:

This joint analysis of a large number of experiments conducted over decades in the Pampas region confirms the importance of body weight at the start of the breeding season to achieve high pregnancy rates in cattle.
