# Supplementary material for: Direct and indirect nutritional factors that determine reproductive performance of heifer and primiparous cows
Source: PLoS One. 2022 Oct 4;17(10):e0275426. doi: 10.1371/journal.pone.0275426 (PMC9531839; doi:10.1371/journal.pone.0275426)
Supplement: Attachment — Submitted filename: Response to Reviewers.doc [file pone.0275426.s004.doc]

PONE-D-21-30153

Direct and indirect nutritional factors that determine reproductive performance of heifer and primiparous cows

PLOS ONE

Dear Academic Editor,

The authors appreciate the comments of the editor and the reviewers, and have accepted the suggestions, which contributed to the improvement of the manuscript.

Changes were made using 'track changes' in MS Word document. Please find below the specific answers to the editor and reviewers’ comments.

**Responses to the Reviewer 4 comments**

Lines 99-101: It is necessary to include “in beef heifers and primiparous cows” in the objective.

Response: The sentence was added and the text was changed to:

The aim of the present study was to integrate information from multiple studies of factors that affect pregnancy rates in beef heifers and primiparous cows under production conditions in the Pampas to carefully quantify response curves relating pregnancy rate to the most important predictors.

Lines 103-104: I think it would be better to use “initial body weight at breeding” and “average daily gains during the breeding season” instead of “body weight” and “changes in body weight per day during breeding season”, respectively.

Response: The sentence was added and the text was changed to:

First, we take an approach where pregnancy rate is analyzed as a function of known proximate factors such as initial body weight at breeding, category and average daily gains during the breeding season.

Lines 106-109: In the hypothesis there’s no mention to factors like: animal category, type of pasture, nor body weight at the beginning of the breeding season. What did the authors expected relative to those factors?

Response: We added the following sentences to the discussion:

Furthermore, body weight at the beginning of the breeding season is expected to have a positive effect on pregnancy rate because of its relationship with physiological status and development. Primiparous cows are expected to have lower pregnancy than heifers due to the physiological stress imposed by recovery from pregnancy and lactation.

Lines 109-111: I think this would be more adequate for the discussion or conclusion section.

Response: The sentence was moved to the Conclusions section, after the first sentence therein.

Line 235: change “with an average weight of 440.0 kg or more and had an expected pregnancy rate of 99.0%” to “with an average weight of 440.0 kg or more had an expected pregnancy rate of 99.0%”.

Response: The word “and” was excluded. The sentence was changed to:

*B. taurus* females starting the breeding season with an average weight of 440.0 kg or more had an expected pregnancy rate of 99.0%, whereas crossbred females that started the breeding season with similar weight had an expected pregnancy rate of 91.0% (Fig 2).

Lines 319-324: This corresponds to the discussion section.

Response: The paragraph was modified as shown below and moved to become the second paragraph in the discussion.

Although body weight at the beginning of the breeding season and average daily gains during the breeding season were dominant in explaining pregnancy rate in the experiments analyzed, multiple factors determine pregnancy rate, many of which are not closely related to body weight, animal category or breed. Other factors explored in some of the experiments analyzed, such as weaning method and use of artificial insemination, were considered and did not have detectable effects in preliminary models for pregnancy rate. However, the different weaning methods and use of artificial insemination were not represented across a good range of values in the other factors.

Lines 468-470: A reference for this would be necessary.

Response: The last sentence was modified and references were added. The last two sentences of the paragraph now read:

When grazing at higher stocking rates, animals are forced to graze closer to the soil and increase the rate of ingestion of parasite helminth larvae (Bransby 1993). Stocking rate might also affect pregnancy rate in ways unrelated to nutrition but linked to the effects on sources of stress related to social interactions among bulls and cows (Fernandez-Novo et al. 2020.

**Responses to the Reviewer 5 comments**

Line 62: From instead of form.

Response: The word “form” was changed to “from”. The phrase was re-written as follows:

An alternative to specific comprehensive studies is to analyze data pooled from multiple studies (Duffield, Merril & Bagg 2012; Lean, Thompson & Dunshea 2014) that address the same research question using equivalent response and explanatory variables, that is, a joint analysis of multiple experiments (Schwarzer, Carpenter & Rücker 2015).

Line 87: Mention the effects of body condition score on pregnancy rate.

Response: The authors added in lines 90 to 92: Body condition score is a critical factor influencing nutritional status of beef cows and determining the success of artificial insemination (Carvalho et al., 2022).

Line 99: Which are your hypothesis ? I recommend to avoid using subjective adverbs such as “carefully”. I assume that if your objective is to quantify, you will do it carefully.

Response: The main hypothesis is in lines 106-109. We removed the word “carefully.”

Line 123: Did you considered the changes in breeds genetics during the period evaluated?

Response: No, we did not. The herds were not subjected to formal selection programs, and each herd was only evaluated one or a few years. We surmise that no relevant directional genetic changes took place in the study.

Line 142: Which were the types of supplements used? Was there a range of supplementation levels?

Response: We included the following table with the information mentioned.

Table 2 – Average and standard error of each variable in the initial database

| Variable | Average |
| --- | --- |
| Age at the beginning of the breeding season^1^ | 24.6±7.5 months |
| Animal categories  Heifers  Primiparous cows | 2257 females  1676 females |
| Body weigth at the beginning of the breeding season | 315.4±55.9 kg |
| Body weigth at the end of the breeding season | 337.3±53.4 kg |
| Breeds  Angus  Braford  Brangus  Crossbred  Devon  Hereford | 306 females  499 females  323 females  1928 females  110 females  767 females |
| Body condition score at the beginning of the breeding season^2^ | 3.2±0.6 |
| Body condition score at the end of the breeding season^2^ | 3.4±0.6 |
| Stocking rate^3^ | 337.32±54.68 kg BW/ha |
| Pasture types  Cultivated  Improved pasture^4^  Natural grassland | 2050  324  1559 |
| Feed supplementation before the breeding season  Not supplemented  Supplemented  Brown rice bran  Commercial concentrate  Corn grain  Deffated rice bran  Deffated rice bran and sorgum silage  Ground corn grain  Sorghum silage and commercial concentrate  Protein salt  Rice and soy bran  Ryegrass and White clover hay  Sectaria hay  Sorghum silage and commercial concentrate | -  0.5 to 1.0% BW  0.7 to 1.5% BW  0.5% BW  1.5% BW  1.5% BW  0.7% BW  1.5% BW  0.1% BW  0.56% BW  0.28% BW  0.92% BW  1.5% BW |

Line 187: Maybe the sentence: “This final model…” sould be change with “the final model after simplifications”, since it may generate confusions with the full model.

Response: The text was changed to:

This final model after simplifications was tested against the full model by a likelihood-ratio test using the anova() function to make sure they were not significantly different.

Line 196: sup.pre was not defined.

Line 202: sup.breed was not defined.

Response: We changed added the information about sup.breed.

The text (lines 201-212) was changed to:

where pasture.pre is a factor indicating whether animals grazed natural grassland, cultivated pastures or improved grassland prior to the breeding period; s.sr is stocking rate, I(s.sr^2) is the quadratic effect of stocking rate, and sup.pre is a binary variable indicating whether animals received supplementation before of the breeding period. Other terms were defined above. Finally, change in body weight during the breeding period (s.dwt) was analyzed starting with the following full model:

s.dwt ~ categ + breed + pasture.breed + s.sr + sup.breed + I(s.sr^2) + pasture.breed:s.sr + categ:s.sr + breed:s.sr + (1 | experiment)

where pasture.breed is the type of pasture grazed during the breeding period and sup.breed is a binary variable indicating whether animals received supplementation during the breeding period.

Line 205: Is sup.pre defined correctly? Or was it confused with sup.breed? Statistical analyses if you reported de means of body condition score of your database, why didn’t include it in your models?

Response: The definitions of sup.pre and sup.breed were added as indicated above.

Line 233: “… 440.0 kg or more and had…” delete the “and”.

Response: Response: The word “and” was excluded. The phrase was changed to:

Pregnancy rate increased steeply with increasing body weight at the beginning of the breeding season for crossbreed and *B. taurus* females, but for *B. taurus* females it increased faster and reached a higher maximum than for crossbreed females. *B. taurus* females starting the breeding season with an average weight of 440.0 kg or more had an expected pregnancy rate of 99.0%, whereas crossbred females that started the breeding season with similar weight had an expected pregnancy rate of 91.0% (Fig 2).

Line 237: Interaction instead of interactin.

Response: The phrase was re-written as follows:

Fig. 2. Interaction between body weight at the beginning of the breeding season and Breed on pregnancy rate of heifers and primiparous cows (Sint: crossbred, Taur: B. Taurus females). Shaded strips represent 95% confidence intervals for the expected value. Body 240 mass average and standard deviation were 318 and 69 kg.

Line 282: Instead of “near its average” I would suggest to use range of daily gain.

Response: Actually, the mathematically correct statement should say “at” the average. We changed the sentence to be:

The response to daily gain was similar to that in the model without stocking rate; pregnancy rate increased 1.5% per 100 g of daily gain when daily gain was at its average.

Line 360: Can you discuss this with your results of body condition score?

Response: We can’t do this because we did not use body condition score in the modeling. We chose to use body weights instead of condition score mainly because of three reasons: (1) body condition score was recorded as a discrete variable with few levels, which prevented its use with traditional gaussian models for quantitative variables, (2) category and body weight together contained most of the information present in body condition score and (3) adding an intermediary “layer” to incorporate the relationships among condition score and the other variables would have complicated the model in a way unnecessary to address our goals. However, we agree that the data could be used for additional exploration of the effects and response of body score, but that would be a different paper.

Line 369: Couldn’t this be corrected by using the body condition score instead of the body weight in the models?

Response: See response above.

Line 393-396: Couldn’t this be corrected by using the body condition score instead of the body weight in the models?

Response: See response above.

Line 436: Correct the citation to “in agreement with Rovira (1974)”.

Response: The citation was changed. The text was changed to:

In agreement with Rovira (1974), we observed that when heifers and primiparous cows are maintained in optimal conditions of grazing and nutrition, that is, maintained in high quality pastures, with sufficient body weight and intermediate stocking rates, they achieve near maximal reproductive success. Sufficient nutrition allows early breeding, which increases the overall efficiency of production for the herd.

Line 452-458: It’s very interesting the analysis of the effects of stocking rate nor mediated by the effects on BW at breeding and BW gain during breeding. You discussed that this effect occurs only when BW at the beginning of the breeding season is greater than average, and mentioned that parasites could be a possible cause of this response. However, parasites mainly affect weight gain. I suggest to discuss which would be the health and management variables that don’t affect the nutritional condition but do affect the reproductive performance.

Response: The authors added in the paragraph: However, herd health, particularly related to infections that directly or indirectly compromise the reproductive tract of females and the embryo and/or fetus, also stands out as an important factor of interference in the reproductive efficiency of beef cattle herds. In free herds, the introduction of the etiologic agent will cause, in most cases, various clinical signs such as repeated estrus, abortion, stillbirth, birth of weak animals and infertility (Junqueira & Alfieri, 2006).

Line 484: be used IN herd reproductive management.

Response: The phrase was changed to:

Body weight at the beginning of the breeding season is an easily measurable variable and can be used as a herd reproductive management tool.

Figure 3: I would suggest to include body mass average and standard deviation as in Figure 2.

Response: We added the following sentence to the caption:

Body mass average and standard deviation were 318 and 69 kg.

Figure 4 and 5: I would suggest to include Stocking rate average and standard deviation as in Figure 2.

Response: We added the following sentence to the caption:

Stocking rate average and standard deviation were 328 and 58.9 kg/ha.

Figure 5: The y axis title is incomplete.

Response: We added the missing closing parenthesis.

Figure 6: This is a great representation, however the size of the figures is too small and it’s difficult to read. The references to the type of line are not clear, and it is hard to distinguish the thick of the lines.

Response: We created a new figure where the individual graphs are larger.
